# Effectiveness of the Project P.A.T.H.S. in Hong Kong: Evaluation Based on Different Strategies and Different Studies over Time

**DOI:** 10.1100/2012/427801

**Published:** 2012-06-04

**Authors:** Daniel T. L. Shek, Hing Keung Ma, Joav Merrick

**Affiliations:** ^1^Department of Applied Social Sciences, The Hong Kong Polytechnic University, Hong Kong; ^2^Public Policy Research Institute, The Hong Kong Polytechnic University, Hong Kong; ^3^Department of Sociology, East China Normal University, Shanghai 200062, China; ^4^Kiang Wu Nursing College of Macau, Macau; ^5^Division of Adolescent Medicine, Department of Pediatrics, Kentucky Children's Hospital, University of Kentucky College of Medicine, Lexington, KY 40506, USA; ^6^Department of Education Studies, Hong Kong Baptist University, Hong Kong; ^7^National Institute of Child Health and Human Development, Jerusalem 91012, Israel

There are worrying trends and phenomena related to the development of adolescents in Hong Kong, such as the intensification of substance abuse and Internet addiction problems [1, 2]. With reference to such adolescent developmental problems, primary prevention programs targeting specific adolescent developmental problems and positive youth development programs are called for. Unfortunately, research findings show that there are very few systematic and multiyear positive youth development programs in Hong Kong. Furthermore, systematic and long-term evaluation of the available youth development programs does not exist [3].

 Against the above background, The Hong Kong Jockey Club Charities Trust approved HK$400 million to launch a project entitled “P.A.T.H.S. to Adulthood: A Jockey Club Youth Enhancement Scheme” to promote adolescent development in junior secondary school students in Hong Kong in 2004. The word “P.A.T.H.S.” denotes Positive Adolescent Training through Holistic Social Programmes. The trust invited academics of five universities in Hong Kong to form a research team with The Hong Kong Polytechnic University as the lead institution to develop a multiyear universal positive youth development program to promote holistic adolescent development in Hong Kong, with the first author as the Principal Investigator. Besides developing the program, the research team also provides training for teachers and social workers who implement the program and carries out longitudinal evaluation of the project. Because of the overwhelming success of the project, it was extended for another cycle with an earmarked grant of HK$350 million in 2008. 

There are two tiers of programs (Tier 1 and Tier 2 Programs) in this project. The Tier 1 Program is a universal positive youth development program in which students in Secondary 1 to Secondary 3 participate, normally with 20 hours of curricular-based training in the school year at each grade. Because research findings suggest that roughly one-fifth of adolescents would need help of a deeper nature, the Tier 2 Program is provided generally for at least one-fifth of the students who have greater psychosocial needs at each grade (i.e., selective program).

The project consists of two implementation phases—the Experimental Implementation Phase and the Full Implementation Phase. For the Experimental Implementation Phase (2005/06 to 2007/08 academic year), 52 secondary schools participated in the project with the objectives of accumulating experience in program implementation and familiarizing frontline workers with the program design and philosophy. In 2006/07 school year, the programs were implemented on a full scale at Secondary 1 level. In 2007/08 school year, the programs were implemented at Secondary 1 and Secondary 2 levels. In 2008/09 school year, the programs were implemented at Secondary 1, Secondary 2 and Secondary 3 levels. For the extension phase of the project, the program would be implemented from 2009/10 to 2011/12 school years.

The overall objective of the Tier 1 Program is to promote holistic development among junior secondary school students in Hong Kong via the inclusion of positive youth development constructs in the program [4]. The positive youth development constructs covered in the Tier 1 Program include promotion of bonding, cultivation of resilience, promotion of social competence, promotion of emotional competence, promotion of cognitive competence, promotion of behavioral competence, promotion of moral competence, cultivation of self-determination, promotion of spirituality, development of self-efficacy, development of a clear and positive identity, promotion of beliefs in the future, provision of recognition for positive behavior, provision of opportunities for prosocial involvement, and fostering prosocial norms. Both Chinese and English curriculum manuals have been published and utilized in the implementation process [4].

Systematic and adequate training is another emphasis of the Project P.A.T.H.S. in Hong Kong [5]. For each of the Secondary 1 to Secondary 3 programs, both teachers and social workers involved receive 20 hours of training before implementing the program in their schools. Generally speaking, there are three days of training at each grade. On day 1, the conceptual foundation, program philosophy, curriculum issues, and evaluation methods are introduced. On day 2 and day 3, the training program covers the teaching units in the curriculum as well as the ways by which the program can be successfully implemented (e.g., program implementers have adequate debriefing skills and adopt reflective practice). In the training program, the potential program implementers are encouraged to reflect on their motivation to teach the program and identification with the program philosophy. They are also empowered to carry out the experiential learning activities that are quite foreign to Chinese teachers. The positive evaluation findings of the training programs have been documented and published [6, 7].

In view of the huge scope of the project, evaluation plays an important role in understanding the impact of the project. Adopting postpositivistic and pragmatic stands where multiple research methods are recognised, different evaluation strategies have been used to evaluate the Tier 1 Program. These strategies include the following

1. *Objective Outcome Evaluation (Randomized Group Trial)*: in the Full Implementation Phase, 24 experimental schools and 24 control schools were randomly selected to participate in a randomized group trial in 2006/07 school year. Analyses of data collected at different time points using individual growth curve models controlling for differences between the two groups in terms of pretest scores, personal variables, and random effects of schools showed that participants in the experimental schools had significantly higher positive youth development levels than did participants in the control schools at posttest based on different indicators derived from the Chinese Positive Youth Development Scale. Students in the experimental schools also displayed less risk behavior than did students in the control schools. The findings based on the total eight waves of data are presented in a paper in this special issue (“*Impact of the Project P.A.T.H.S. in the junior secondary school years: objective outcome evaluation based on eight waves of longitudinal data*” by D. T. L. Shek and C. M. S. Ma; “*Longitudinal impact of the Project P.A.T.H.S. on adolescent risk behavior: what happened after five years?*” by D. T. L. Shek and L. Yu).
2. *Subjective Outcome Evaluation (Tier 1 Program)*: both students and program implementers were invited to complete subjective outcome evaluation forms (Form A and Form B, resp.) after completion of the program to understand their perceptions of the program, the implementers and benefits of the program. Existing quantitative as well as qualitative findings generally showed that different stakeholders had positive views about the program and the program was beneficial to the participants. The subjective outcome evaluation findings were found to converge with objective outcome evaluation findings and the related changes. In the present special issue, two papers based on the perceptions of the program participants and implementers are included (“*Participants' evaluation of the Project P.A.T.H.S.: are findings based on different datasets consistent?*” by D. T. L. Shek and R. C. F. Sun; “*Program implementers' evaluation of the Project P.A.T.H.S.: findings based on different datasets over time*” by D. T. L. Shek and C. M. S. Ma).
3. *Subjective Outcome Evaluation (Secondary Data Analyses)*: to gain an in-depth understanding of the impact of the project, program implementers were invited to write down five conclusions regarding the project based on the Form A and Form B data. In separate studies, secondary data analyses of the conclusions drawn by the program implementers based on the Form A and Form B reports revealed that different stakeholders had positive views of the program and they perceived the program to be beneficial to the program participants. Integration of the secondary data analyses of the reports in all cohorts of students in the project revealed that the program was perceived to be beneficial to the program participants. The detailed findings can be seen in the paper in this special issue (“*Secondary data analyses of subjective outcome evaluation data based on nine databases*” by D. T. L. Shek).
4. *Process Evaluation*: in process evaluation, systematic observations were carried out by trained research assistants in randomly selected schools to understand the program implementation details. Several studies generally revealed that program adherence was high, with a mean adherence of over 80%. Besides, the findings generally showed that the program implementation quality in these schools was high. In this special issue, an integrative study analyzing all process evaluation data collected at different stages of the project was carried out. The findings showed that the overall program adherence and implementation quality was high (“*Process evaluation of a positive youth development program in Hong Kong based on different cohorts*” by B. M. F. Law and D. T. L. Shek).
5. *Interim Evaluation*: to understand the process of implementation, interim evaluation was conducted by randomly interviewing roughly half of the participating schools in the Experimental Implementation Phase or the Full Implementation Phase. In this special issue, the data in all interim evaluation studies were integrated and analyzed. The findings generally showed that the participants and implementers perceived the merits and benefits of the program, although difficulties in implementing the program and recommendations for improving the program and the implementation process were observed (“*Interim evaluation of the Project P.A.T.H.S.: findings based on different datasets*” by D. T. L. Shek and L. Yu Interim).
6. *Qualitative Evaluation (Focus Groups Based on Students)*: focus groups involving students based on schools randomly selected from the participating schools were carried out in previous years. In this special issue, all focus group data based on previous studies were subject to secondary data analyses. With specific focus on how the informants described the program, results showed that the descriptors used were mainly positive in nature. When the informants were invited to name three metaphors that could stand for the program, the related metaphors were basically positive in nature. Finally, the program participants perceived many beneficial effects of the program in different psychosocial domains (“*Qualitative evaluation of the Project P.A.T.H.S.: an integration of findings based on program participants*” by D. T. L. Shek and R. C. F. Sun).
7. *Qualitative Evaluation (Focus Groups Based on Program Implementers)*: focus groups involving program implementers based on schools randomly selected from the participating schools were also conducted in the project. Secondary data analyses of the related data collected in the project revealed several observations. First, the program implementers identified strengths and positive features of the program. Second, they perceived the program to be beneficial to the development of the program participants. Third, they proposed suggestions on how the program could be improved. Generally speaking, the program implementers have positive evaluation of the program (“*Qualitative evaluation of the Project P.A.T.H.S.: an integration of findings based on program implementers*” by D. T. L. Shek).
8. *Evaluation Based on Student Weekly Diaries*: after completion of the Tier 1 Program, students were randomly selected from the participating schools to write a reflective journal in the form of weekly diary to reveal their perceptions and feelings regarding the Tier 1 Program and the related benefits. Secondary data analyses showed that most of the respondents had positive views on the program, had positive views on the instructors, and stated that they had acquired competencies at the societal, familial, interpersonal, and personal levels after joining the program (“*Evaluation of the Project P.A.T.H.S. based on students' weekly diaries: findings from eight datasets*” by D. T. L. Shek and R. C. F. Sun).
9. *Evaluation Based on Repertory Grid Test*: at the end of the Full Implementation Phase, students were randomly selected from schools to complete repertory grid tests to understand how the participants perceived changes in their identity at different points of time. The findings generally showed that the participants had improved self-representations after joining the program (“*Evaluation of a positive youth development program based on the repertory grid test*” by D. T. L. Shek).
10. *Subjective Outcome Evaluation (Tier 2 Program)*: the participants were invited to complete subjective outcome evaluation forms (Form C) after completion of the Tier 2 Program to understand their perceptions of the program, the implementers, and benefits of the program. Existing quantitative as well as qualitative findings generally showed that different stakeholders had positive views about the program, the implementers, and benefits of the program. In the present special issue, one paper based on the perceptions of the program participants about the Tier 2 Program is included (“*Helping adolescents with greater psychosocial needs: subjective outcome evaluation based on different cohorts*” by D. T. L. Shek and T. Y. Lee).

There are several special features of the studies covered in this special issue. First, large sample sizes were involved in different evaluation studies. For example, 206,313 Tier 1 Program participants responded to the subjective outcome evaluation form (Form A), 7,926 Tier 1 Program implementers responded to the subjective outcome evaluation form (Form B), 60,241 Tier 2 Program participants responded to subjective outcome evaluation form (Form C), and 1,138 pieces of student diaries were collected. Second, data based on different cohorts of participants were integrated and analyzed in different studies covered in the papers reported in this special issue. The use of different cohorts can give an aggregated picture of evaluation findings over time. Third, based on the wide range of evaluation strategies used in the studies included in this special issue, the findings consistently showed that different stakeholders had positive perceptions of the Project P.A.T.H.S. and the two tiers of program were beneficial to the development of the program participants. Finally, this is the first known collection of evaluation studies of a positive youth development program in different Chinese contexts based on multiple evaluation strategies and longitudinal data (“*Positive youth development programs in Chinese communities: where are we and where should we go?*” by D. T. L. Shek and R. C. F. Sun) [8–10]. It is our modest wish that through the project and the related evaluation studies, evidence-based positive youth development programs can be promoted in different Chinese communities [11, 12].
